# Supplementary material for: Predicting the Efficacy and Safety of TACTICs (Tumor Angiogenesis-Specific CAR-T Cells Impacting Cancers) Therapy for Soft Tissue Sarcoma Patients
Source: Cancers (Basel). 2020 Sep 23;12(10):2735. doi: 10.3390/cancers12102735 (PMC7598286; doi:10.3390/cancers12102735)
Supplement: Supplementary file 1 [file cancers-12-02735-s001.pdf]

Supplementary Materials

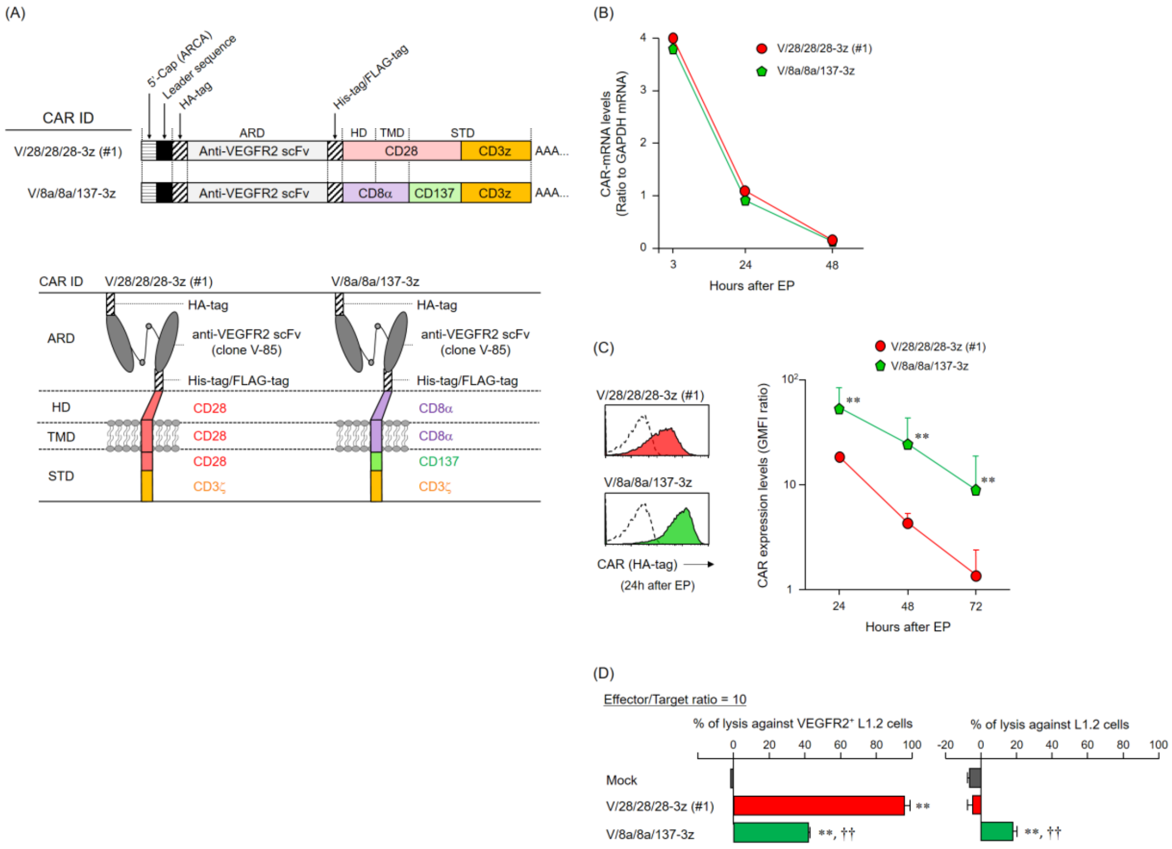

Figure S1. CAR expression profile and VEGFR2-specific cytotoxic activity of human T cells transfected with different CAR variants via the EP method. (**A**) Illustration of VEGFR2-specific CARs. The upper panel represents the mRNA constructs containing the VEGFR2-specific second-generation CAR genes; the lower panel represents the expected CAR proteins. (**B**) Profile of CAR-mRNAs introduced into human T cells. CAR-mRNAs introduced into human T cells were analyzed using reverse transcription-quantitative polymerase chain reaction. CAR-mRNA expression is represented, relative to that of GAPDH-mRNA. The data are shown as the mean ± SD of triplicates and are representative of at least two independent experiments. Statistical analysis was performed using the Student's *t*-test and showed no significant differences. (**C**) Expression profiles of CAR proteins on T cell membranes. CAR expression was analyzed using flow cytometry using anti-HA-tag (solid color histograms) or isotype control antibodies (dashed white histograms). CAR expression levels are given by the ratio between anti-HA-tag mAb staining-GMFI and isotype control antibody staining-GMFI. The histograms on the left are representative results of CAR expression levels, obtained 24 h after EP. The data on the right shows the mean ± SD of three individual experiments. Statistical analysis was performed using the Student's *t*-test: ** $p < 0.01$. (**D**) In vitro cytotoxic activity of human CAR-T cells. Human CAR-T cells cultured for 24 h after EP were co-cultured with L1.2 cells and VEGFR2$^+$ L1.2 cells at an E/T ratio of 10 for 18 h. Then, the number of L1.2 and VEGFR2$^+$ L1.2 cells in the wells was evaluated using flow cytometry. The cytotoxic activity against VEGFR2$^+$ L1.2 cells was calculated from the ratio between the number of VEGFR2$^+$ L1.2 cells in the context of CAR-T versus mock-T cells. The cytotoxic activity against L1.2 cells was also calculated from the ratio between the number of L1.2 cells in the context of CAR-T versus mock-T cells. The data are represented as the mean ± SD of the three individual experiments using different donor-derived T cells. Statistical analysis was performed using the Tukey's test: **$p < 0.01$, CAR-T cells versus mock-T cells; ††$p < 0.01$, CAR [V/8a/8a/137-3z]-T cells versus CAR [V/28/28/28-3z (#1)]-T cells.
